# Airway management in pre-hospital critical care: a review of the evidence for a ‘top five’ research priority

**DOI:** 10.1186/s13049-018-0556-4

**Published:** 2018-10-20

**Authors:** K. Crewdson, M. Rehn, D. Lockey

**Affiliations:** 1 Department of Anaesthesia, North Bristol NHS Trust, Southmead Hospital, Southmead Way, Bristol, BS10 5NB UK; 2 Department of Research, Norwegian Air Ambulance Foundation, Drøbak, Norway; 3 Pre-hospital Division, Air Ambulance Department, Oslo University Hospital, Oslo, Norway; 4 Faculty of Health Sciences, University of Stavanger, Stavanger, Norway; 5 Bristol University, Bristol, UK

**Keywords:** Airway management, Emergency medical services, Intubation

## Abstract

The conduct and benefit of pre-hospital advanced airway management and pre-hospital emergency anaesthesia have been widely debated for many years. In 2011, prehospital advanced airway management was identified as a ‘top five’ in physician-provided pre-hospital critical care. This article summarises the evidence for and against this intervention since 2011 and attempts to address some of the more controversial areas of this topic.

## Background

Pre-hospital emergency anaesthesia (PHEA) and advanced airway management remains a controversial subject. There are mixed views about whether advanced interventions are beneficial or detrimental [1–4]. What is clear however, is that there is a small but identifiable group of patients with recognised indications for intubation, in whom basic airway manoeuvers are not sufficient to maintain adequate oxygenation, and advanced airway interventions are warranted at an early stage [5].

The quality of pre-hospital emergency airway management has progressed significantly. For many years intubation was usually only performed for patients in cardiac arrest or in those with an absent gag reflex, and was associated with a poor outcome [6, 7]. The number of advanced airway interventions performed in the pre-hospital setting has increased significantly over recent years. A meta-analysis published in 2010 reported a total of 54,933 intubation attempts [8]. In a subsequent meta-analysis from 2006 to 2016, 125,177 intubation attempts were reported [9]. It is likely that one of the major factors contributing to these findings is the increasing number of physicians involved in pre-hospital care, particularly in European practice. This observation is borne out by further data from meta-analyses which reported only 127 intubation attempts by physicians up to and including 2009 [8], compared with 23,738 intubation attempts by physicians between 2006 and 2016 [9]. The recent interest in pre-hospital advanced airway management has generated more research in this area but the majority of studies are single centre retrospective database reviews, with significant heterogeneity in the design, methodology and endpoints, making interpretation and the generation of meaningful conclusions difficult [10, 11]. Randomised controlled trials are difficult to conduct in a pre-hospital or major trauma setting due to issues around consent and inclusion criteria [12, 13].

In 2011, an expert consensus process identified advanced airway management as one of the top five research priorities in pre-hospital critical care [10]. Some of the most important questions remain the most difficult to answer, for example, what are the indications for pre-hospital advanced airway management, does it confer a survival benefit, which patients should receive it, who should deliver it? [10]. The aim of this article is to present the arguments for and against the practice of advanced pre-hospital airway management and PHEA and attempt to address some of the controversy surrounding this topic.

### Is pre-hospital advanced airway management necessary?

One method of addressing whether advanced airway interventions performed in the pre-hospital setting are actually necessary is to assess whether there is an unmet need for pre-hospital intubation. This can be determined by identifying the number of patients who arrive in the emergency department (ED) with indications for urgent tracheal intubation. Relatively few studies directly address this issue and those that do suggest that there is an unmet demand for urgent tracheal intubation for a proportion of trauma patients in whom basic airway manoeuvres are inadequate. One study from a pre-hospital physician-led service based in the United Kingdom (UK) investigated trauma patients who required any airway interventions. Despite the presence of ambulance personnel, 57% of patients still had airway compromise on arrival of the physician-led trauma team. All patients required emergency intubation on scene [5]. Unpublished data obtained by the author (KC) from the UK trauma audit and research network also suggests an unmet demand in the delivery of pre-hospital advanced airway management. In the United States, approximately 10% of trauma admissions require advanced airway intervention within the first 5 hours of hospital arrival; over half of the patients had indications for urgent intubation including reduced level of consciousness, hypoventilation or hypoxaemia, or airway obstruction [14, 15].

### Which patients need it?

Emergency airway management in any setting has a significant risk of complications; [16] complication rates of up to 13% have been reported [17], and careful selection of the correct patients is part of improving the overall success of the procedure. There are some indications which require immediate airway intervention, including complete airway obstruction, failure to oxygenate or ventilate adequately, cardiac arrest or a Glasgow Coma Scale (GCS) less than 9 [18]. In some circumstances, basic airway techniques may provide temporary management of oxygenation and ventilation but advanced airway techniques are usually required to provide definitive airway control. There is considerable variation in the indications for pre-hospital emergency airway management. Datasets produced from analysis of United States (US) airway registries are often difficult to interpret but, of the two largest published recently, one study from 2011 reports 10,356 intubation attempts [19] and one from 2014 reports 74,993 intubation attempts [20]. The 2011 data from Wang et al. suggest that the major indication for intubation is cardiac arrest and this is supported by the 2014 data from Diggs et al., with the studies reporting that 53 and 52% of patients respectively are intubated following a cardiac arrest. The frequency of intubation following trauma is also similar between the two studies - 6 and 8% [19, 20]. Outside the US, one of the largest pre-hospital emergency airway studies of the last 5 years comes from Sunde et al. reporting data on 2327 intubations from multiple centres. In this study, 55% of patients were intubated for medical reasons, of whom 62% were in cardiac arrest. The remaining 45% of patients are intubated following traumatic injury, of who 56% are in cardiac arrest [21].

There are circumstances in which early intubation may not be in the patient’s best interests. There is data to suggest that patients with significant hypovolaemia following traumatic injury may have a higher mortality if anaesthetised in the pre-hospital setting, and that shorter scene times and waiting until arrival at hospital with direct access to definitive surgical intervention may be preferable for this patient group [22, 23].

### How should it be done?

The question of the optimal techniques for pre-hospital airway management remain much debated. The majority of studies in this area focus on out-of-hospital cardiac arrest and only a small number describe techniques used for trauma patients. Some studies which compare the use of bag-valve mask (BVM) ventilation with advanced airway management techniques found no benefit of advanced airway techniques over BVM ventilation [3, 24] but other studies do suggest a morbidity and mortality benefit associated with the use of advanced airway techniques for all severely-injured patients [25–27] and for those patients with traumatic brain injury if performed by personnel with appropriate training and experience [4, 28, 29]. Those studies that focus on out-of-hospital cardiac arrest also do not conclusively show a benefit of advanced techniques over basic techniques but the inability to adjust for confounders is widely acknowledged [30–32]. A UK-based study assessing the use of supraglottic airway devices for non-traumatic out-of-hospital cardiac arrest failed to demonstrate superiority when compared with tracheal intubation [33]. In contrast, data from the United States suggested improved 72-h survival using supraglottic airway devices when compared to tracheal intubation [34]. Data published in 2018 from a trial comparing bag-valve-mask ventilation with tracheal intubation for initial airway management was inconclusive [35].

PHEA is usually performed using an induction agent, often ketamine, a neuromuscular blocker and a sedative agent. As with in-hospital practice, the majority of agents can be safely used in a pre-hospital setting as long as careful attention is given to the dose of drug administered, to reflect the deranged physiology of severely-injured patients. The use of ketamine as an induction agent has historically been associated with an increase in intracranial pressure [36]. More recent studies suggest these concerns are not associated with any clinical significance and ketamine is now considered a safe and effective drug for use in the pre-hospital setting [37], particularly in haemodynamically unstable patients [38]. Rocuronium is the neuromuscular blocking agent of choice for many and a combination of fentanyl, ketamine, and rocuronium has been shown to produce more favourable intubating conditions in the pre-hospital setting [39].

All efforts should be focused on making the first attempt at laryngoscopy successful, as repeated attempts have been shown to be detrimental both in terms of morbidity and mortality [40, 41]. Multiple attempts at laryngoscopy can cause bleeding or swelling in the airway and may result in significant desaturation and hypoxic episodes [42]. Laryngoscopy is highly stimulating for patients and causes a sympathetic surge. Perkins et al. demonstrated a hypertensive response to pre-hospital laryngoscopy and intubation in 79% of severely injured patients, and 9% of patients experienced a greater than 100% increase in mean arterial pressure and/or systolic blood pressure [43]. Impairment of cerebral autoregulation following traumatic brain injury leaves the brain vulnerable to surges in blood pressure and intracranial pressure, with a subsequent worsening of cerebral oedema and haematoma expansion, which can be detrimental to patient outcome [43–45]. The hypertensive response to laryngoscopy is arguably more common in emergency settings, where the dose of induction agent may be modified if there are significant concerns about the severity of injury and the likely physiological response to anaesthesia. Opioids which suppress the hypertensive response, may be given in low doses or omitted altogether.

A robust failed intubation plan should be well-embedded into all services delivering PHEA. This plan should be verbalised to the attending team before starting PHEA. Videolaryngoscopy may be considered as part of a failed intubation plan or may at times be used for the first attempt at laryngoscopy. The benefit of videolaryngoscopy for emergency airway management remains widely debated but recent evidence does not strongly support a positive benefit of this intervention [46–48]. Emergency cricothyroidotomy is generally the endpoint of failed intubation guidelines [49, 50]. The evidence-base for this technique remains small and no clear benefit of a surgical technique has been demonstrated over needle technique however the increased number of complications associated with needle cricothyroidotomy and the requirement for conversion to a surgical technique means that a surgical technique is recommended by major airway guidelines [49, 51].

### Who should deliver it?

There is ongoing debate about who should deliver pre-hospital advanced airway management and the amount of training required and consensus has not been reached. Recommendations in recent UK guidelines suggest that the standard of care delivered in the pre-hospital setting should be the same as that delivered in-hospital and doctors providing emergency anaesthesia should be able to do so competently, and unsupervised, in the emergency department [50, 52]. Recognition of Pre-hospital Emergency Medicine (PHEM) as a subspecialty in the UK has helped structure and formalise training programmes in pre-hospital care to improve the care delivered to patients. In Europe, pre-hospital emergency care is increasingly delivered by physicians [53]. There is evidence to suggest higher success rates and shorter on scene times for PHEA when this technique is delivered by physicians [54]. A median intubation success rate of 98.8% (range 78.1–100%) has been reported for physicians performing intubations in the pre-hospital setting. The reported median success rate for non-physicians is 91.7% (range 61.6 to 100%) [9]. As expected, success rates are generally higher for anaesthetists when compared with non-anaesthetists [53, 55], emphasising the importance of increased clinical exposure in the preservation of skills, and avoidance of skill fade [56]. In recognition of the fact that intubation without the use of drugs is generally futile [6], the Joint Royal Colleges Ambulance Liaison Committee no longer train paramedics in tracheal intubation but recommend the use of supraglottic airway devices for advanced airway management [57].

### How can practice be improved?

#### Standards and safety

PHEA has become increasingly formalised and guidelines exist at local and national levels to standardise the procedure and improve patient safety [50, 52, 58]. The pre-hospital infrastructure in the United States differs significantly from that in Europe and Australasia and although the guidelines reflect those differences, the general messages delivered are similar in all the guidelines. There is a strong focus on patient safety, the guidelines suggest that advanced airway management should only be delivered when appropriately skilled pre-hospital personnel are available. Otherwise meticulous attention should be paid to performing high-quality basic airway interventions [50, 52, 58, 59]. Studies which have reviewed the implementation and effectiveness of these tools within pre-hospital services have been able to demonstrate uncomplicated introduction process [60] and improvement in compliance with guideline standards [61, 62].

#### Apnoeic oxygenation

Severely injured patients with significant physiological and anatomical derangement, are more susceptible to adverse events during emergency anaesthesia. Anatomical distortion of the head and neck from injury may impede intubation, and chest injury may cause ventilation-perfusion mismatch. Hypoxia is one of the most commonly occurring adverse event during emergency intubation and is reported to occur in over one quarter of emergency intubations [63, 64]. Whilst any given reduction in the partial pressure of arterial oxygen will reduce arterial oxygen saturation, the magnitude of this fall increases once SaO_2_ falls below 93% [65]. Increasing the time to desaturation during prolonged or difficult intubation using apnoeic oxygenation has been demonstrated to increase peri-intubation oxygen saturation and reduce the incidence of hypoxaemia. The technique, though simple to perform, remains relatively underused in the pre-hospital setting. One retrospective study reported a 6% reduction in episodes of desaturation associated with emergency intubations [66]. Further studies are being conducted to evaluate its use in the pre-hospital environment.

#### Post intubation care

In line with in-hospital practice, there is an increasing focus on post-intubation care. If possible, post intubation care should begin in the pre-hospital phase. Patients should be appropriately sedated using an anaesthetic agent following intubation, the dose of which is titrated to their haemodynamic physiology. Further doses of neuromuscular blocking agents may also be required to enable mandatory ventilation and avoid any ventilatory compromise. The use of end-tidal carbon dioxide monitoring, has become mandatory in any intubated patient and careful attention should be paid to the provision of appropriate ventilation strategies, incorporating lung protective ventilation if possible. Emerging evidence about the harmful effects of hyperoxia may guide future practice, particularly in patients with traumatic brain injury where a PaO_2_ greater than 65 kPa (or 487 mmHg) has been shown to worsen patient outcome [67]. Ventilation should be carefully managed to avoid hypocarbia and hypercarbia, both of which have been demonstrated to be detrimental, particularly in traumatic brain injury [68–70]. Mechanical ventilation is generally considered to be superior to hand ventilation when targeting a specific range for end-tidal carbon dioxide [71]. One Scandinavian service demonstrated increased use of mechanical ventilation following the introduction of an standard operating procedure [62].

Body temperature should be maintained in the pre-hospital setting. Recent data has demonstrated a higher rate of hypothermia in patients who are anaesthetised outside hospital [62]. Previously cooling patients with traumatic brain injury or post cardiac arrest was considered to be beneficial to outcome but subsequent studies have questioned this theory and it is no longer recommended practice [72, 73].

#### Reporting data

The standardised reporting of data for pre-hospital advanced airway management remains poor despite recent major guidelines promoting the use of key performance indicators [52]. In 2009, Sollid et al. developed an Utstein-style template for documenting and reporting pre-hospital airway management [11], but to date its use remains limited with relatively few studies reporting data in accordance with the template. The template has been recently revised [74] and improvements in data collection and reporting will make the evidence-base for pre-hospital advanced airway management more robust and provide better indications of the benefits and pitfalls of this intervention.

## Conclusion

PHEA remains a controversial area with a limited evidence-base but current data suggests an unmet demand for PHEA in a small but identifiable group of patients. Where necessary, the intervention should be delivered by personnel with the appropriate skills and training. Careful attention should be given to optimising the first attempt at laryngoscopy and the intervention should be delivered to the same standards as those achieved in hospital. The increasing numbers of physicians in Pre-hospital Emergency Medicine should help improve the delivery of PHEA, which will hopefully translate into improvement in morbidity and mortality.

## Abbreviations

BVM: Bag-valve-mask
GCS: Glasgow Coma Scale
PaO_2_: Arterial partial pressure of oxygen
PHEA: Pre-hospital Emergency Anaesthesia
PHEM: Pre-hospital Emergency Medicine
